# Assessing individual equivalence in parallel group and crossover designs: Exact test and sample size procedures

**DOI:** 10.1371/journal.pone.0269128

**Published:** 2022-05-27

**Authors:** Gwowen Shieh

**Affiliations:** Department of Management Science, National Yang Ming Chiao Tung University, Hsinchu, Taiwan; University Politehnica of Bucharest, ROMANIA

## Abstract

The consideration of individual equivalence provides an essential alternative to average equivalence in two-group comparative studies. A common procedure for declaring individual equivalence adopts the tolerance intervals of the designated proportions of measurement differences. This statistical practice is a direct generalization of the widely used two one-sided tests (TOST) for average equivalence. Such TOST extensions often do not have adequate control of Type I error and result in excessively conservative tests. To signify and resolve the underlying issues of existing methods, this paper presents exact tests for assessing individual equivalence between two treatments under parallel group and crossover designs. Rigorous evaluations are conducted to clarify the discrepancy of critical values and Type I error probabilities between the equivalence procedures. The findings elucidate the shortcoming of the TOST technique and the advantage of the proposed approach. The associated power and sample size calculations are also justified through simulation studies.

## Introduction

The two one-sided tests (TOST) procedure of mean equivalence, first described by Schuirmann [1] and Westlake [2], is the most common method in equivalence methodology. The conceptual simplicity and technical feasibility of TOST provide an important reform to apply appropriate statistical tools for equivalence, rather than relying on failure to reject the conventional hypothesis of no difference between treatment effects. Meyners [3] presented a comprehensive review of different types of equivalence tests. Moreover, Hauschke, Steinijans, and Pigeot [4], Chow and Liu [5], Wellek [6], and Choudhary and Nagaraja [7] discussed the concepts and techniques for the design and analysis of equivalence studies. The TOST for mean equivalence focuses on the mean parameters of the target populations and represents a vital method within the general scope of average equivalence. It is important to note that mean equivalence testing specifies only the population mean difference and does not concern the other characteristics associated with the underlying distribution of measurement differences. Accordingly, the principle of average equivalence only demands similar average bioavailability and does not guarantee equivalence in intra-subject variability and closeness of the response distribution between the test and reference formulations.

In view of the practical issue and important problem about the interchangeability of bioequivalence drug products, the notion of individual equivalence has been proposed to ensure switchability when a large proportion of individuals need to be sufficiently similar on the two drug formulations. The basic concept and rationale of individual equivalence are described in Anderson and Hauck [8], Hauck and Anderson [9], Sheiner [10], Schall and Luus [11], and Anderson [12]. Various individual equivalence principles and techniques have been proposed to evaluate exchangeability or switchability in terms of the desired proportion of the subject-level differences between two formulations. In particular, the commonly used reference limits of 95% proportion encompass the 2.5th percentile and 97.5th percentile for the distribution of measurement differences. Accordingly, the normal percentile is a linear function of the mean and standard deviation of the designated population. Statistical procedures and theoretical investigations of normal percentiles are essential for assessing individual equivalence.

For the mean equivalence appraisals considered in the TOST, the duality between decision rules and confidence intervals is well documented. Specifically, the null hypothesis of no mean equivalence is rejected if and only if the confidence limits of the corresponding equal-tail two-sided 100(1–2α)% confidence interval of mean difference are contained in the designated equivalence bounds. Within the context of individual equivalence, the target parameters are the lower and upper percentiles for describing the desired population proportion. It is appealing to apply the confidence interval procedure to the normal percentiles of the distribution of subject-level differences. The one-sided confidence intervals of normal percentiles have a close link to the one-sided tolerance bounds of a normal distribution. This technical correspondence reveals that tolerance interval estimation has an extended utility in assessing individual bioequivalence. The notion of confidence intervals for mean equivalence or average equivalence has been extended to the appraisals of individual equivalence, such as the TOST methods presented in Esinhart and Chinchilli [13], Liu and Chow [14], and Tsong and Shen [15], among others. Accordingly, tolerance intervals are constructed for the desired proportions of measurement differences and individual equivalence is claimed if the resulting interval limits are within the selected equivalence range. General discussions of tolerance interval estimation are available in Krishnamoorthy and Mathew [16] and Meeker, Hahn, and Escobar [17].

Due to the close resemblance between tolerance intervals and confidence intervals, the TOST method for assessing individual equivalence is presumed to share the same desirable properties of the counterpart TOST for establishing mean equivalence. However, Berger and Hsu [18] showed that size-α bioequivalence tests do not generally correspond to 100(1–2α)% confidence sets. It is strongly advocated in Berger and Hsu [18] that statistically sound techniques should be employed to derive a test with the specified Type I error rate. Notably, the prescribed TOST methods for individual equivalence were conducted with respect to tolerance interval estimation. The corresponding numerical results did not directly evaluate their Type I error control in hypothesis testing. Although the assessment of individual equivalence mainly focuses on biopharmaceutical applications, the concept and analysis are pertinent to comparative studies across virtually all scientific disciplines. It is of great interest to clarify the potential deficiency and implications of current methods in equivalence testing.

Following the two-sided sampling plan in Owen [19], this article presents a unified approach for evaluating individual equivalence between two treatment formulations. Exact test procedures are described for the parallel group and crossover designs. Extensive numerical investigations are conducted to demonstrate the underlying features the suggested and TOST procedures. The comparisons and findings reveal their essential discrepancy on critical values and Type I error rates that have not been addressed in the literature. The results update the less-recognized problems of the current TOST methods for examining individual equivalence in Liu and Chow [14], and Tsong and Shen [15]. To enhance the usefulness of the proposed approach, the associated power and sample size calculations are also demonstrated for planning individual equivalence studies. Computer algorithms for computing the critical value, statistical power, and sample size of the suggested test procedures are available as supplemental material. It should be noted that Owen [19] did not address hypothesis testing, power analysis and sample size determination for appraising individual equivalence. Moreover, the technical arguments presented here are more analytically transparent than the formulation based on the bivariate noncentral *t* distribution in Owen [19].

## Methods

### Parallel group design

Consider independent random samples from two normal populations with the following formulations:

$$X_{ij}\sim N(\mu_{i},\sigma^{2}),$$ (1)

where μ_*i*_, σ^2^ are unknown parameters, *j* = 1, …, *N*_*i*_, and *i* = 1 and 2. To establish individual equivalence between two treatments, the central portion of the difference between the individual measurements of two treatments *X*_1*j*_–*X*_2*j′*_ needs to lie within a reasonable range around zero. The 100·*p*th percentile of the distribution *N*(μ_*D*_, $\sigma_{D}^{2}$) of *X*_1*j*_–*X*_2*j′*_ is denoted by

$$\theta_{p}=\mu_{D}+z_{p}\sigma_{D},$$ (2)

where μ_*D*_ = μ_1_– μ_2_, $\sigma_{D}^{2}$ = 2σ^2^, *z*_*p*_ is the 100·*p*th percentile of the standard normal distribution *N*(0, 1), and 0 < *p* < 1. The null and alternative hypotheses of the individual equivalence test are expressed as

$${\text{H}}_{0}:\theta_{1-p}\leq \;\Delta_{L}\text{or}\;\Delta_{U}\leq \theta_{p}\;\text{versus}\;{\text{H}}_{1}:\;\Delta_{L}\;<\;\theta_{1-p}\;\text{and}\;\theta_{p}\;<\;\Delta_{U},$$ (3)

where *p* > 0.5 and the two designated constants Δ_*L*_ and Δ_*U*_ represent the lower and upper thresholds of the percentile range for declaring individual equivalence between two treatments. The alternative hypothesis indicates that there is at least p* = 2*p* – 1 central proportion of the distribution *N*(μ_*D*_, $\sigma_{D}^{2}$) in the range (Δ_*L*_, Δ_*U*_).

Unlike the individual equivalence problem concerns the central proportion of a target distribution in terms of the pair of percentiles (θ_1-*p*_, θ_*p*_), a comparison of alternative approaches for difference, noninferiority, and equivalence testing of a single normal percentile was presented in Shieh [20]. Similar to the widely used TOST for mean equivalence, Shieh [20] showed that the TOST procedure for the comparability of a designated percentile also maintains good control the Type I error rate at the specified value. These promising results suggest that TOST principle can be useful for similar problems in more advanced designs and complex scenarios. However, a critical exposition of the TOST extensions for individual equivalence is presented to demonstrate that such generalizations do not have adequate control of Type I error and result in overly conservative tests.

#### The TOST procedure for parallel group design

To demonstrate average equivalence between two treatment means, the TOST procedure rejects the null hypothesis of incomparability if the ordinary 100(1–2α)% equal-tailed confidence interval of mean difference is entirely included in the equivalence range. The same principle was extended to individual equivalence assessment for exchangeability between the test and standard treatments in Tsong and Shen [15]. A concise illustration is presented to simplify the complicated results in Tsong and Shen [15].

The usual two-sample *t* statistic has the form

$$T=\frac{{\bar{X}}_{1}-{\bar{X}}_{2}}{{\left(S^{2}/M\right)}^{1/2}},$$

where ${\bar{X}}_{1}=\sum_{j=1}^{N_{1}}X_{1j}/N_{1}$, ${\bar{X}}_{2}=\sum_{j=1}^{N_{2}}X_{2j}/N_{2}$, *M* = 1/(1/*N*_1_ + 1/*N*_2_), *S*^2^ = {(*N*_1_−1) $S_{1}^{2}$ + (*N*_2_−1) $S_{2}^{2}$}/*v*, $S_{1}^{2}=\sum_{j=1}^{N_{1}}{\left(X_{1j}-{\bar{X}}_{1}\right)}^{2}/\left(N_{1}-1\right)$, $S_{2}^{2}=\sum_{j=1}^{N_{2}}{\left(X_{2j}-{\bar{X}}_{2}\right)}^{2}/\left(N_{2}-1\right)$, and *v* = *N*_1_ + *N*_2_−2. The ordinary interval limits (${\hat{\mu }}_{DL}$, ${\hat{\mu }}_{DU}$) of a 100(1–2α)% equal-tailed confidence interval of μ_*D*_ are

$${\hat{\mu }}_{DL}=({\bar{X}}_{1}-{\bar{X}}_{2})-t_{\nu ,\;1-\alpha }\left(S/M^{1/2}\right)\;\text{and}\;{\hat{\mu }}_{DU}=({\bar{X}}_{1}-{\bar{X}}_{2})+t_{\nu ,\;1-\alpha }(S/M^{1/2}),$$ (4)

respectively, where *t*_*v*,1−*α*_ is the 100(1 – α)th percentile of the *t* distribution with degrees of freedom *v*. In addition to the practical usefulness for interval estimation, the range {${\hat{\mu }}_{DL}$, ${\hat{\mu }}_{DU}$} has an interesting connection to equivalence assessment. A well-known simple approach to conduct the TOST for mean equivalence is by examining whether the 100(1–2α)% confidence interval (${\hat{\mu }}_{DL}$, ${\hat{\mu }}_{DU}$) of μ_*D*_ falls within the designated range (δ_*L*_, δ_*U*_) where δ_*L*_ and *δ*_*U*_ are a priori constant and represent the sensible bounds for declaring mean equivalence.

It is straightforward to show that the pivotal quantity for θ_1-*p*_ has a noncentral *t* distribution

$$\frac{{\bar{X}}_{1}-{\bar{X}}_{2}-\theta_{1-p}}{{\left(S^{2}/M\right)}^{1/2}}∼t\left(\nu ,\;z_{p}{\left(2M\right)}^{1/2}\right),$$ (5)

where *t*(*v*, *z*_*p*_(2*M*)^1/2^) is a noncentral *t* distribution with degrees of freedom ν and noncentrality *z*_*p*_(2*M*)^1/2^. The exact lower confidence limit of an upper 100(1 – α)% one-sided confidence interval {${\hat{\theta }}_{TL}$, ∞} of θ_1−*p*_ can be obtained as

$${\hat{\theta }}_{TL}={\bar{X}}_{1}-{\bar{X}}_{2}-\tau_{T}\left(S/M^{1/2}\right),$$ (6)

where τ_*T*_ = *t*_1−*α*_(*v*, *z*_*p*_(2*M*)^1/2^) is the 100(1 – α)th percentile of a noncentral *t* distribution *t*(*v*, *z*_*p*_(2*M*)^1/2^). Similarly, the pivotal quantity for θ_*p*_ is distributed as

$$\frac{{\bar{X}}_{1}-{\bar{X}}_{2}-\theta_{p}}{{\left(S^{2}/M\right)}^{1/2}}∼t\left(\nu ,\;-z_{p}{\left(2M\right)}^{1/2}\right).$$ (7)

Using the important property of a noncentral distribution as in Johnson, Kotz, and Balakrishnan [21, Chapter 31] that *t*_1−*α*_(*v*, *z*_*p*_(2*M*)^1/2^) = −*t*_*α*_(*v*, −*z*_*p*_(2*M*)^1/2^) for 0 < α < 1, the exact upper confidence limit of a lower 100(1 – α)% two-sided confidence interval {–∞, ${\hat{\theta }}_{TU}$} of θ_*p*_ can be expressed as

$${\hat{\theta }}_{TU}={\bar{X}}_{1}-{\bar{X}}_{2}+\tau_{T}\left(S/M^{1/2}\right).$$ (8)

Note that the one-sided confidence intervals of normal percentiles are technically identical to the one-sided tolerance bounds of a normal distribution as noted in Hahn [22, 23]. The derived confidence limits ${\hat{\theta }}_{TL}$ and ${\hat{\theta }}_{TU}$ assure that *P*{${\hat{\theta }}_{TL}$ < θ_1−*p*_ < ∞} = *P*{*P*[${\hat{\theta }}_{TL}$ < (*X*_1*j*_–*X*_2*j′*_) | (${\bar{X}}_{1}$ – ${\bar{X}}_{2}$, *S*^2^)] > *p*} = 1 – α and *P*{–∞ < θ_*p*_ < ${\hat{\theta }}_{TU}$} = *P*{*P*[(*X*_1*j*_–*X*_2*j′*_) < ${\hat{\theta }}_{TU}$ | (${\bar{X}}_{1}$ – ${\bar{X}}_{2}$, *S*^2^)] > *p*} = 1 – α, respectively. Accordingly, for *p* > 0.5, a lower 100(1 – α)% confidence limit for the 100(1–*p*)-th percentile θ_1−*p*_ is equivalent to a lower tolerance limit to be exceeded by at least a proportion *p* of the population with probability 1 – α. Likewise, an upper 100(1 – α)% confidence limit for the 100 *p*-th percentile θ_*p*_ for *p* > 0.5 is equivalent to an upper tolerance limit to exceed at least a proportion *p* of the population with probability 1 – α.

As an extension to the use of tolerance intervals for the assessment of individual bioequivalence, Tsong and Shen [15] suggested that the null hypothesis H_0_: θ_1−*p*_ ≤ Δ_*L*_ or Δ_*U*_ ≤ θ_*p*_ is rejected if

$$\Delta_{L}<{\hat{\theta }}_{TL}\;\text{and}\;{\hat{\theta }}_{TU}<\Delta_{U},$$ (9)

or

$$T_{L}=\frac{{\bar{X}}_{1}-{\bar{X}}_{2}-\Delta_{L}}{{\left(S^{2}/M\right)}^{1/2}}>\tau_{T}\;\text{and}\;T_{U}=\frac{{\bar{X}}_{1}-{\bar{X}}_{2}-\Delta_{U}}{{\left(S^{2}/M\right)}^{1/2}}<-\tau_{T}.$$ (10)

The strong resemblance between (${\hat{\mu }}_{DL}$, ${\hat{\mu }}_{DU}$) and {${\hat{\theta }}_{TL}$, ${\hat{\theta }}_{TU}$} in formulation and testing suggests that the rejection region {${\hat{\theta }}_{TL}$, ${\hat{\theta }}_{TU}$} for individual equivalence may possess similar statistical properties with the confidence interval (${\hat{\mu }}_{DL}$, ${\hat{\mu }}_{DU}$) for mean equivalence. Specifically, the TOST of mean equivalence based on (${\hat{\mu }}_{DL}$, ${\hat{\mu }}_{DU}$) adequately controls the Type I error rate at the specified value. However, Berger and Hsu [18] exemplified that an equivalence procedure in terms of a 100(1–2α)% confidence interval can lead to a liberal or conservative test. The Type I error rate associated with the TOST of individual equivalence is evaluated by α_*TOST*_ = *P*{τ_*T*_ < *T*_*L*_ and *T*_*U*_ < −τ_*T*_} when the boundary values (θ_1−*p*_, θ_*p*_) = (Δ_*L*_, Δ_*U*_). It follows from Δ_*L*_ = θ_1−*p*_ = μ_*D*_−*z*_*p*_σ_*D*_ and Δ_*U*_ = θ_*p*_ = μ_*D*_ + *z*_*p*_σ_*D*_ that *T*_*L*_ ~ *t*(*v*, *z*_*p*_(2*M*)^1/2^) and *T*_*U*_ = *T*_*L*_− 2*z*_*p*_σ_*D*_/(*S*^2^/*M*)^1/2^. Thus, the Type I error rate is rewritten as α_*TOST*_ = *P*{τ_*T*_ < *T*_*L*_ and *T*_*U*_ < −τ_*T*_} = *P*{τ_*T*_ < *T*_*L*_ < 2*z*_*p*_σ_*D*_/(*S*^2^/*M*)^1/2^ – τ_*T*_} ≤ *P*{τ_*T*_ < *T*_*L*_} = α. Note that the size of the TOST is the supremum $\begin{array}{c} Sup\\ H_{0} \end{array}P\left\{\Delta_{L}\leq {\hat{\theta }}_{TL}\;and\;{\hat{\theta }}_{TU}\leq \Delta_{U}\right\}$ = α which is attained as $\sigma_{D}^{2}$ or σ^2^ goes to zero. However, the Type I error rate of the TOST procedure is generally less than the nominal level. The succeeding empirical investigations reveal that the discrepancy is of considerable concern. An improved procedure is proposed next to facilitate research practice in assessing individual equivalence.

#### The proposed procedure for parallel group design

By extending the two-sided sampling plan in Owen [19], the suggested exact rejection region for declaring individual equivalence is of the form

$$\Delta_{L}<{\hat{\theta }}_{EL}\;\text{and}\;{\hat{\theta }}_{EU}<\Delta_{U}$$ (11)

where ${\hat{\theta }}_{EL}={\bar{X}}_{1}-{\bar{X}}_{2}+\tau_{E}(S/M^{1/2})$, ${\hat{\theta }}_{EU}={\bar{X}}_{1}-{\bar{X}}_{2}+\tau_{E}(S/M^{1/2})$, and the quantity τ_*E*_ is selected so that the Type I error rate $\begin{array}{c} Sup\\ H_{0} \end{array}P\left\{\Delta_{L}\leq {\hat{\theta }}_{EL}\;and\;{\hat{\theta }}_{EU}\leq \Delta_{U}\right\}$ = α. Note that the supremum $\begin{array}{c} Sup\\ H_{0} \end{array}P\left\{\Delta_{L}\leq {\hat{\theta }}_{EL}\;and\;{\hat{\theta }}_{EU}\leq \Delta_{U}\right\}$ is attained when the two percentiles coincide the boundary values (θ_1−*p*_, θ_*p*_) = (Δ_*L*_, Δ_*U*_) or alternatively, μ_*D*_ = (Δ_*U*_ + Δ_*L*_)/2 and $\sigma_{D}^{2}$ = (Δ_*U*_− Δ_*L*_)^2^/(4${\text{z}}_{p}^{2}$). Accordingly, the designated critical value τ_*E*_ is obtained by

$$P(\theta_{1-p}\leq {\hat{\theta }}_{EL}\;\text{and}\;{\hat{\theta }}_{EU}\leq \theta_{p})=\alpha .$$ (12)

It follows from the normal assumption defined in Eq 1 that *Z* = (${\bar{X}}_{1}$ – ${\bar{X}}_{2}$ – μ_*D*_)/(σ^2^/*M*)^1/2^ ~ *N*(0, 1) and *K* = *vS*^2^/σ^2^ ~ χ^2^(*v*) where χ^2^(*v*) is a chi-square distribution with degrees of freedom *v*. Also, *Z* and *K* are independent. Then, the probability evaluation in Eq 12 can be expressed as

$$P\left(–G\leq Z\leq G\right)=\alpha ,$$ (13)

where *G* = *z*_*p*_(2*M*)^1/2^ – τ_*E*_(*K*/*v*)^1/2^. It is computationally transparent to adopt the formulation

$$E_{K}[2\Phi \left(G_{0}\right)\;–1]=\alpha ,$$ (14)

where *G*_0_ = *G* if *K* < *k*_0_ and *G*_0_ = 0 if *K* ≥ *k*_0_ with *k*_0_ = (2*vM${\text{z}}_{p}^{2}$*)/$\tau_{E}^{2}$, Φ is the cumulative density function of the standard normal distribution, and the expectation *E*_*K*_[·] is taken with respect to the distribution of *K*. A special-purpose computer program is required to calculate the critical value τ_*E*_ for the chosen model settings. Consequently, the null hypothesis is rejected if

$$T_{L}>\tau_{E}\;\text{and}\;T_{U}\;<\;–\tau_{E}.$$ (15)

Note that the critical values τ_*E*_ of the suggested approach and τ_*T*_ of the TOST procedure generally differ. For example, when (*N*_1_, *N*_2_) = (20, 20), α = 0.05, p* = 0.80, the critical values are τ_*E*_ = 6.4527 and τ_*T*_ = 7.9987 for the suggested and TOST procedures, respectively. According to the rejection rules in Eqs 10 and 15, the TOST is less likely to reject the null hypothesis than the exact procedure because of τ_*T*_ > τ_*E*_. Therefore, the two critical regions (${\hat{\theta }}_{EL}$, ${\hat{\theta }}_{EU}$) and (${\hat{\theta }}_{TL}$, ${\hat{\theta }}_{TU}$) do not necessarily lead to the same conclusion.

On the other hand, with the definitions of the two random variables *Z* and *K*, it can be shown that the corresponding power function is

$$\Psi_{E}=P\left(G_{L}<Z<G_{U}\right),$$ (16)

where *G*_*L*_ = (Δ_*L*_− μ_*D*_)/(σ^2^/*M*)^1/2^ + τ_*E*_(*K*/*v*)^1/2^ and *G*_*U*_ = (Δ_*U*_− μ_*D*_)/(σ^2^/*M*)^1/2^ – τ_*E*_(*K*/*v*)^1/2^. Note that the power calculation is meaningful only when *G*_*L*_ < *G*_*U*_ or *K* < *k*_1_ where *k*_1_ = {*vM*(Δ_*U*_− Δ_*L*_)^2^}/(4$\sigma^{2}\tau_{E}^{2}$). A transparent and convenient expression of the power function is

$$\Psi_{E}=E_{K}[\Phi \left(G_{U}{}_{1}\right)-\Phi \left(G_{L}{}_{1}\right)],$$ (17)

where *G*_*L*1_ = *G*_*L*_ and *G*_*U*1_ = *G*_*U*_ if *K* < *k*_1_, and *G*_*L*1_ = 0 and *G*_*U*1_ = 0 if *K* ≥ *k*_1_. The power formula Ψ_*E*_ is useful for computing the achieved power with the given sample sizes, and for determining the required sample sizes to attain the nominal power under the selected configurations (Δ_*L*_, Δ_*U*_, p*, α, μ_1_, μ_2_, σ^2^).

### Crossover design

In bioequivalence studies, a common scenario for comparing treatments is the two-period crossover design. Consider the standard two-sequence and two-period crossover design in terms of the model

$$Y_{ijk}{}_{}=\mu +F_{ij}+P_{j}+S_{ik}+\varepsilon_{ijk}$$ (18)

where *Y*_*ijk*_ is the outcome for the *k*th subject in the *i*th sequence and *j*th period, μ is the grand mean, *F*_*ij*_ is the formulation effect, *P*_*j*_ is the fixed period effect, *S*_*ik*_ is the random subject effect, and *ε*_*ijk*_ is the random error for *i* = 1 and 2, *j* = 1 and 2, and *k* = 1, …, *N*_*i*_. The formulation effects are expressed as *F*_11_ = *F*_22_ = μ_*R*_ and *F*_12_ = *F*_21_ = μ_*T*_ for the reference product and test product, respectively, {*S*_*ik*_} are independent *N*(0, $\sigma_{S}^{2}$) variables, and {ε_*ijk*_} are independent *N*(0, $\sigma_{ij}^{2}$) variables with $\sigma_{11}^{2}$ = $\sigma_{22}^{2}$ = $\sigma_{R}^{2}$ and $\sigma_{12}^{2}$ = $\sigma_{21}^{2}$ = $\sigma_{T}^{2}$. Moreover, it is assumed that *P*_1_ + *P*_2_ = μ*R* + μ_*T*_ = 0.

To establish individual equivalence between two treatments in the crossover design, the central portion of the contrast for the individual measurements of two treatments (*C*_1*k*_–*C*_2*k′*_)/2 needs to be within a reasonable range around zero where *C*_*ik*_ = (*Y*_*i*2*k*_–*Y*_*i*1*k*_)/2 for *i* = 1 and 2. Accordingly, (*C*_1*k*_–*C*_2*k′*_) ~ *N*(μ_*C*_, $\sigma_{C}^{2}$) where μ_*C*_ = μ_*T*_− μ_*R*_, $\sigma_{C}^{2}$ = 2σ^2^, and σ^2^ = ($\sigma_{R}^{2}$ + $\sigma_{T}^{2}$)/4. The 100·*p*th percentile for the distribution of (*C*_1*k*_–*C*_2*k′*_) is denoted by

$$\theta_{p}=\mu_{C}+z_{p}\sigma_{C}$$ (19)

for 0 < *p* < 1 as in Eq 2. An unbiased estimator of the difference between the two treatments μ_*C*_ is the sample mean difference ${\bar{C}}_{1}-{\bar{C}}_{2}$ where ${\bar{C}}_{1}$ = $\sum_{k=1}^{N_{i}}C_{ik}/N_{i}$ for *i* = 1 and 2. It is clear that *E*(${\bar{C}}_{1}$) = μ_*C*_/2, *E*(${\bar{C}}_{2}$) = –μ_*C*_/2, *Var*(${\bar{C}}_{1}$) = σ^2^/*N*_1_, and *Var*(${\bar{C}}_{2}$) = σ^2^/*N*_2_. Hence, the mean difference ${\bar{C}}_{1}$ – ${\bar{C}}_{2}$ has the distribution

$${\bar{C}}_{1}-{\bar{C}}_{2}∼N(\mu_{C},\;\sigma^{2}/M),$$

where *M* = 1/(1/*N*_1_ + 1/*N*_2_). Moreover, *S*^2^ = $\sum_{i=1}^{2}\sum_{k=1}^{N_{i}}{\left(C_{ik}-{\bar{C}}_{i}\right)}^{2}/\nu$ is an unbiased estimator of σ^2^ and *K* = (*vS*^2^)/σ^2^ has a chi-square distribution with degrees of freedom *v* = *N*_1_ + *N*_2_−2. The formulations and properties for the crossover design show close resemblance to those of the parallel group design. Accordingly, the conceptual and statistical similarities enable the conversion of the individual equivalence inference of the parallel group design into that of the crossover design.

#### The TOST procedure for crossover design

By analogy to the parallel group design, the individual equivalence problem within the context of crossover design can be conducted with respect to the null and alternative hypotheses given in Eq 3. Following the TOST principle for assessing equivalence of mean effects, Liu and Chow [14] proposed an extension for declaring individual equivalence based on the lower confidence limit of a upper 100(1 – α)% one-sided confidence interval of θ_1−*p*_ and the upper confidence limit of a lower 100(1 – α)% one-sided confidence interval of θ_*p*_. Specifically, Liu and Chow [14] suggested that the null hypothesis of no individual equivalence is rejected if

$$\Delta_{L}<{\hat{\theta }}_{CTL}\;\text{and}\;{\hat{\theta }}_{CTU}<\Delta_{U},$$ (20)

or

$$T_{CL}=\frac{{\bar{C}}_{1}-{\bar{C}}_{2}-\Delta_{L}}{{\left(S^{2}/M\right)}^{1/2}}\;>\;\tau_{CT}\;\text{and}\;T_{CU}=\frac{\;{\bar{C}}_{1}-{\bar{C}}_{2}-\Delta_{U}}{{\left(S^{2}/M\right)}^{1/2}}\;<\;-\tau_{CT}.$$ (21)

where ${\hat{\theta }}_{CTL}={\bar{C}}_{1}-{\bar{C}}_{2}–\tau_{CT}\left(S/M^{1/2}\right)$, ${\hat{\theta }}_{CTU}={\bar{C}}_{1}-{\bar{C}}_{2}+\tau_{CT}\left(S/M^{1/2}\right)$ and the critical value τ_*CT*_ = τ_*T*_ = *t*_1−*α*_(*v*, *z*_*p*_(2*M*)^1/2^).

#### The proposed procedure for crossover design

In this case of crossover design, the proposed exact rejection region for declaring individual equivalence is of the form

$$\Delta_{L}<{\hat{\theta }}_{CEL}\;\text{and}\;{\hat{\theta }}_{CEU}<\Delta_{U}$$ (22)

where ${\hat{\theta }}_{CEL}={\bar{C}}_{1}-{\bar{C}}_{2}–\tau_{CE}\left(S/M^{1/2}\right)$, ${\hat{\theta }}_{CEL}={\bar{C}}_{1}-{\bar{C}}_{2}+\tau_{CE}\left(S/M^{1/2}\right)$, and the quantity τ_*CE*_ is selected so that the Type I error rate $\begin{array}{c} Sup\\ H_{0} \end{array}P\left\{\Delta_{L}\leq {\hat{\theta }}_{CEL}\;and\;{\hat{\theta }}_{ECU}\leq \Delta_{U}\right\}$ = α. This evaluation of the Type I error rate has the same statistical property as that of the parallel group design. The critical value can be obtained with the identical technique. Consequently, with the similar argument and notation, it can be shown that the critical value τ_*CE*_ is identical to that of the parallel group design: τ_*CE*_ = τ_*E*_. Alternatively, the null hypothesis is rejected if

$$T_{CL}>\tau_{CE}\;\text{and}\;T_{CU}<-\tau_{CE}.$$ (23)

The corresponding power function is

$$\Psi_{CE}=P\left\{G_{CL}<Z<G_{CU}\right\},$$ (24)

where *G*_*CL*_ = (Δ_*L*_− μ_*C*_)/(σ^2^/*M*)^1/2^ + τ_*CE*_(*K*/*v*)^1/2^, *G*_*CU*_ = (Δ_*U*_− μ_*C*_)/(σ^2^/*M*)^1/2^ – τ_*CE*_(*K*/*v*)^1/2^, *Z* ~ *N*(0, 1), and *K* ~ χ^2^(*v*). For computational ease, an alternative formulation of Ψ_*CE*_ is

$$\Psi_{CE}=E_{K}[\Phi \left(G_{CU}{}_{1}\right)–\Phi \left(G_{CL}{}_{1}\right)],$$ (25)

where *G*_*CL*1_ = *G*_*CL*_ and *G*_*CU*1_ = *G*_*CU*_ if *K* < *k*_*C*1_, *G*_*CL*1_ = 0 and *G*_*CU*1_ = 0 if *K* ≥ *k*_*C*1_, *k*_*C*1_ = {*vM*(Δ_*U*_− Δ_*L*_)^2^}/(${4\sigma }^{2}\tau_{CE}^{2}$). For ease of illustration, the endpoints of the prescribed test procedures for parallel group and crossover designs are summarized in Table 1.

**Table 1:** The endpoints of the proposed and TOST rejection rules.

Methods	Endpoints	Equation
The TOST procedure by Tsong and Shen [15]: {${\hat{\theta }}_{TL}$, ${\hat{\theta }}_{TU}$}	$\begin{array}{l} {\hat{\theta }}_{TL}={\bar{X}}_{1}-{\bar{X}}_{2}-\tau_{T}\left(S/M^{1/2}\right)\\ {\hat{\theta }}_{TU}={\bar{X}}_{1}-{\bar{X}}_{2}+\tau_{T}\left(S/M^{1/2}\right) \end{array}$	9
The proposed procedure: {${\hat{\theta }}_{EL}$, ${\hat{\theta }}_{EU}$}	$\begin{array}{l} {\hat{\theta }}_{EL}={\bar{X}}_{1}-{\bar{X}}_{2}-\tau_{E}\left(S/M^{1/2}\right)\\ {\hat{\theta }}_{EU}={\bar{X}}_{1}-{\bar{X}}_{2}+\tau_{E}(S/M^{1/2}) \end{array}$	11
The TOST procedure by Liu and Chow [14]: {${\hat{\theta }}_{CTL}$, ${\hat{\theta }}_{CTU}$}	$\begin{array}{l} {\hat{\theta }}_{CTL}={\bar{C}}_{1}-{\bar{C}}_{2}-\tau_{CT}\left(S/M^{1/2}\right)\\ {\hat{\theta }}_{CTU}={\bar{C}}_{1}-{\bar{C}}_{2}+\tau_{CT}\left(S/M^{1/2}\right) \end{array}$	20
The proposed procedure: {${\hat{\theta }}_{CEL}$, ${\hat{\theta }}_{CEU}$}	$\begin{array}{l} {\hat{\theta }}_{CEL}={\bar{C}}_{1}-{\bar{C}}_{2}-\tau_{CE}(S/M^{1/2})\\ {\hat{\theta }}_{CEU}={\bar{C}}_{1}-{\bar{C}}_{2}+\tau_{CE}(S/M^{1/2}) \end{array}$	22

## Results

### Type I errors

The suggested test procedures are derived by controlling the Type I error at the nominal level. Although the critical values do not have an explicit analytic expression, they can be determined with the designated configurations (*N*_1_, *N*_2_, p*, α, Δ_*L*_, Δ_*U*_). On the other hand, the TOST procedures generalize the results for mean equivalence assessment and tolerance interval estimation. The resulting critical values and rejection regions are not directly obtained with respect to the Type I error control in hypothesis testing. It is of theoretical and practical importance to evaluate the potential discrepancy between the proposed approach and benchmark TOST method. Accordingly, simulation study was conducted to examine the Type I error rates under the parallel group designs.

For the numerical investigations, the selected central proportions of the individual equivalence tests are p* = 0.80, 0.90 and 0.95. The mean and variance of the null distribution *N*(μ_*D*0_, $\sigma_{D0}^{2}$) for the individual measurement difference are chosen as μ_*D*0_ = 0 and $\sigma_{D0}^{2}$ = 1. The designated thresholds (Δ_*L*_, Δ_*U*_) are determined by Δ_*L*_ = μ_*D*0_–*z*_*p*_σ_*D*0_ and Δ_*U*_ = μ_*D*0_ + *z*_*p*_σ_*D*0_. The resulting similarity bounds are (Δ_*L*_, Δ_*U*_) = (–1.2816, 1.2816), (–1.6449, 1.6449), and (–1.9600, 1.9600) for *p* = 0.90, 0.95, and 0.975, respectively. Four sets of sample sizes are considered: (*N*_1_, *N*_2_) = (20, 20), (50, 50), (100, 100), and (200, 200). Throughout the empirical examination, the significance level is fixed as α = 0.05. Under the combined twelve structures of central proportions and sample sizes, an important step is to compute the critical values τ_*E*_ and τ_*T*_ of the proposed and TOST procedures for the specified settings. According to the results presented in Table 2, the two critical values have a systematic order that τ_*E*_ is consistently less than τ_*T*_. Hence, the TOST method has smaller rejection rate than the suggested approach.

**Table 2:** The critical values of the proposed and TOST procedures for individual equivalence when the significance level α = 0.05.

		Sample sizes (*N*_1_, *N*_2_)			
Test procedure	Central proportion p*	(20, 20)	(50, 50)	(100, 100)	(200, 200)
The proposed approach	0.80	6.4527	9.7099	13.4337	18.7232
TOST method		7.9987	11.1886	14.8840	20.1553
The proposed approach	0.90	8.4041	12.5728	17.3474	24.1334
TOST method		9.8812	13.9793	18.7236	25.4901
The proposed approach	0.95	10.1084	15.0664	20.7517	28.8354
TOST method		11.5352	16.4203	22.0744	30.1377

**Fig 1:**
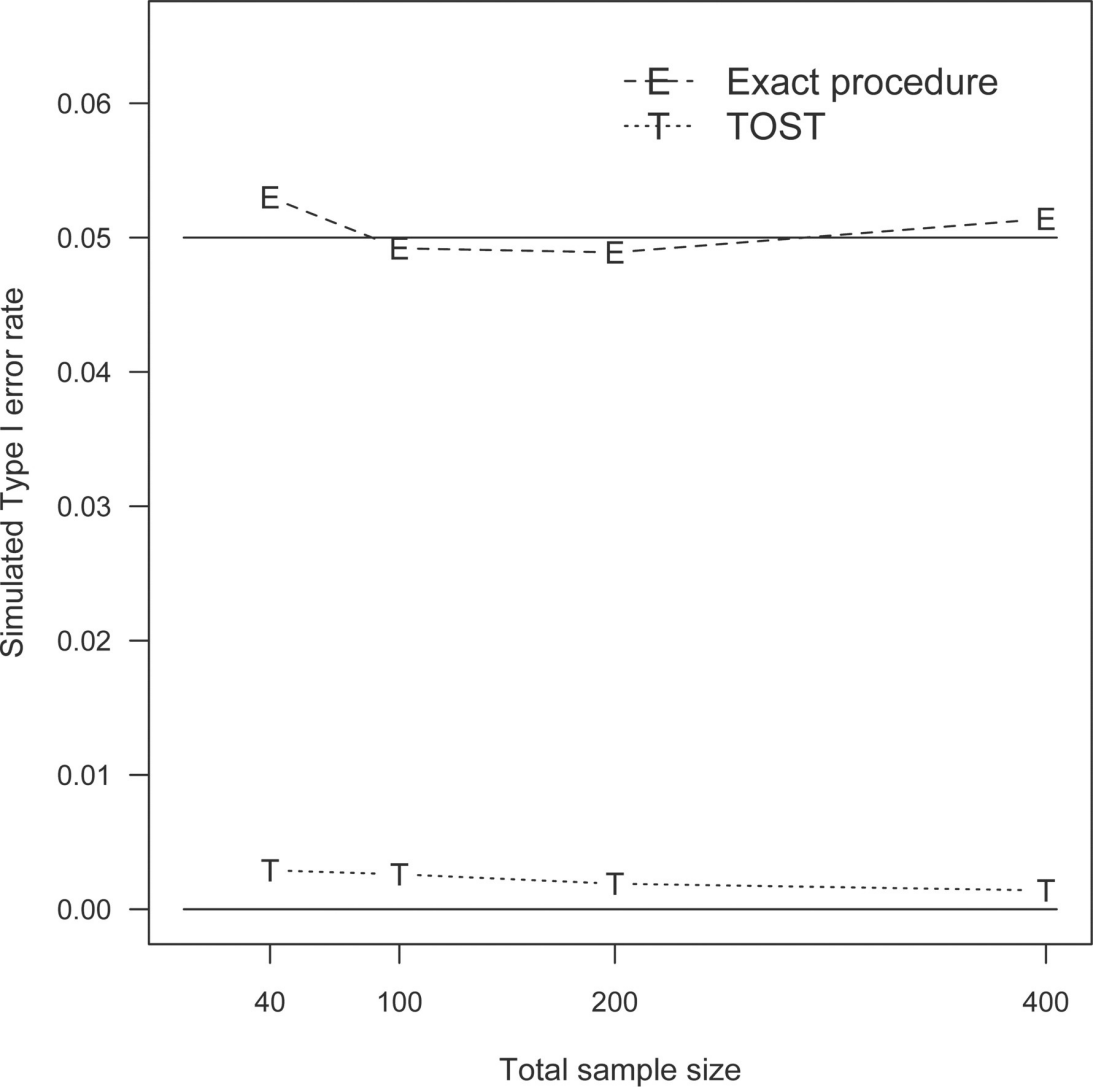
Simulated Type I error rates for central proportion 0.90 and α = 0.05.

The simulated Type I error rates of the individual equivalence tests were computed via Monte Carlo simulation of 10,000 independent data sets. For the two test procedures, the simulated Type I error rates were the proportion of the 10,000 replicates whose critical intervals (${\hat{\theta }}_{EL}$, ${\hat{\theta }}_{EU}$) and (${\hat{\theta }}_{TL}$, ${\hat{\theta }}_{TU}$) were within the range of (Δ_*L*_, Δ_*U*_). The simulated Type I error probabilities under the four different sample sizes are summarized in Tables 3–5 for the three central portions p* = 0.80, 0.90, and 0.95, respectively. The adequacy of the two procedures is determined by the difference between the simulated Type I error rate and the nominal level 0.05 as summarized in the tables. To visualize the differences between the two procedures, the simulated results for p* = 0.90 in Table 4 are also plotted in Fig 1. It is evident that the simulated Type I error rates of the suggested approach are almost identical to the nominal value 0.05. In contrast, the simulated Type I error probabilities of the TOST method are less than 0.01 for the 12 settings considered here. These findings suggest that the proposed procedure has adequate Type I error control, whereas the TOST procedure is extremely conservative.

**Table 3:** The simulated Type I error rates of individual equivalence tests for central proportion p* = 0.80, equivalence bounds (Δ_*L*_, Δ_*U*_) = (–1.2816, 1.2816), and the significance level α = 0.05.

	Sample sizes (*N*_1_, *N*_2_)							
	(20, 20)		(50, 50)		(100, 100)		(200, 200)	
Test procedure	Simulated alpha	Difference	Simulated alpha	Difference	Simulated alpha	Difference	Simulated alpha	Difference
The proposed approach	0.0541	0.0041	0.0486	–0.0014	0.0506	0.0006	0.0496	–0.0004
TOST procedure	0.0011	–0.0489	0.0008	–0.0492	0.0004	–0.0496	0.0004	–0.0496

Note: Δ_*L*_ = μ_*D*_−*z*_*p*_σ_*D*_ and Δ_*U*_ = μ_*D*_ + *z*_*p*_σ_*D*_ where μ_*D*_ = 0, $\sigma_{D}^{2}$ = 1, *p* = 0.90, and *z*_*p*_ = 1.2816.

**Table 4:** The simulated Type I error rates of individual equivalence tests for central proportion p* = 0.90, equivalence bounds (Δ_*L*_, Δ_*U*_) = (–1.6449, 1.6449), and the significance level α = 0.05.

	Sample sizes (*N*_1_, *N*_2_)							
	(20, 20)		(50, 50)		(100, 100)		(200, 200)	
Test procedure	Simulated alpha	Difference	Simulated alpha	Difference	Simulated alpha	Difference	Simulated alpha	Difference
The proposed approach	0.0530	0.0030	0.0492	–0.0008	0.0489	–0.0011	0.0514	0.0014
TOST procedure	0.0029	–0.0471	0.0026	–0.0474	0.0019	–0.0491	0.0014	–0.0486

Note: Δ_*L*_ = μ_*D*_−*z*_*p*_σ_*D*_ and Δ_*U*_ = μ_*D*_ + *z*_*p*_σ_*D*_ where μ_*D*_ = 0, $\sigma_{D}^{2}$ = 1, *p* = 0.95, and *z*_*p*_ = 1.6449.

**Table 5:** The simulated Type I error rates of individual equivalence tests for central proportion p* = 0.95, equivalence bounds (Δ_*L*_, Δ_*U*_) = (–1.9600, 1.9600), and the significance level α = 0.05.

	Sample sizes (*N*_1_, *N*_2_)							
	(20, 20)		(50, 50)		(100, 100)		(200, 200)	
Test procedure	Simulated alpha	Difference	Simulated alpha	Difference	Simulated alpha	Difference	Simulated alpha	Difference
The proposed approach	0.0518	0.0018	0.0502	0.0002	0.0493	–0.0007	0.0522	0.0022
TOST procedure	0.0056	–0.0444	0.0041	–0.0459	0.0032	–0.0468	0.0031	–0.0469

Note: Δ_*L*_ = μ_*D*_−*z*_*p*_σ_*D*_ and Δ_*U*_ = μ_*D*_ + *z*_*p*_σ_*D*_ where μ *=* = 0, $\sigma_{D}^{2}$ = 1, *p* = 0.975, and *z*_*p*_ = 1.9600.

### Power and sample size calculations

A related and important issue of the individual equivalence test is the power and sample size calculations. The power functions derived in Eqs 17 and 25 facilitate the desired power and sample size planning of the parallel group and crossover designs. The algorithms for computing the critical value, achieved power, and sample size are implemented in the supplementary programs. Accordingly, numerical studies were conducted to explicate the behavior of derived power function and the usefulness of accompanying computer algorithm in sample size determinations.

Sample size determination requires test configurations of Type I error rate α, nominal power 1 – β, equivalence bounds (Δ_*L*_, Δ_*U*_), null central portion p*, and the alternative settings include the mean values (μ_1_, μ_2_), error variance σ^2^, and sample size allocation ratio *r* = *N*_2_/*N*_1_. Note that the resulting percentiles θ_1−*p*_ and θ_*p*_ need to be within the designated bounds (Δ_*L*_, Δ_*U*_) under the alternative distribution *N*(μ_*D*_, $\sigma_{D}^{2}$). For illustration, two central portions are considered: p* = 0.90 and 0.95 (*p* = 0.95 and 0.975). By fixing the null distribution *N*(μ_*D*_, $\sigma_{D}^{2}$) as *N*(0, 1), the resulting two sets of threshold bounds are (Δ_*L*_, Δ_*U*_) = (–1.6449, 1.6449), and (–1.9600, 1.9600). The alternative distributions are chosen to have the treatment means (μ_1_, μ_2_) = (0, 0), (0.05, 0), and (0.10, 0), and variance $\sigma_{D}^{2}$ = 0.6, 0.7 and 0.8. Under the specified configurations, the minimum total sample size *N*_*T*_ = *N*_1_ + *N*_2_ is computed for balanced design *r* = 1 (*N*_1_ = *N*_2_), significance level α = 0.05, and nominal power 1 – β = 0.9. The estimated sample sizes and attained power levels are summarized in Table 6 for the combined 18 cases. The minimum sample size for attaining the nominal power increases with increasing mean difference μ_*D*_ or increasing variance $\sigma_{D}^{2}$ when all other factors remain fixed. It is essential to see that the magnitudes of the computed sample sizes are substantially different for the settings considered here. The smallest sample size is 80 for two the settings of (p*, μ_*D*_, $\sigma_{D}^{2}$) = (0.95, 0, 0.6). On the other hand, the largest sample size 1852 is required for the situation with (p*, μ_*D*_, $\sigma_{D}^{2}$) = (0.90, 0.10, 0.8). The results indicate that the prescribed test configurations have unique and distinct influence on the power function. Conceivably, it is unlikely that a simple guideline will give accurate sample size determination.

**Table 6:** Estimated sample size, estimated power, and simulated power of the proposed individual equivalence test for balanced design *N*_1_ = *N*_2_, σ^2^ = $\sigma_{D}^{2}$/2, the nominal power 0.90, and the significance level α = 0.05.

Null proportion p*	Equivalence bounds (Δ_*L*_, Δ_*U*_)	Mean μ_*D*_	Variance $\sigma_{D}^{2}$	Sample size *N*_*T*_	Simulated power	Estimated power	Difference
0.90	(–1.6449, 1.6449)	0	0.6	86	0.9020	0.9008	0.0012
			0.7	182	0.8973	0.9004	–0.0031
			0.8	482	0.9034	0.9009	0.0025
		0.05	0.6	92	0.9026	0.9005	0.0021
			0.7	210	0.9019	0.9020	–0.0001
			0.8	678	0.9012	0.9005	0.0007
		0.10	0.6	116	0.9013	0.9027	–0.0014
			0.7	322	0.8961	0.9005	–0.0044
			0.8	1852	0.9032	0.9001	0.0031
0.95	(–1.9600, 1.9600)	0	0.6	80	0.8981	0.9006	–0.0025
			0.7	168	0.9036	0.9007	0.0029
			0.8	440	0.9029	0.9003	0.0026
		0.05	0.6	86	0.9075	0.9057	0.0018
			0.7	186	0.9021	0.9008	0.0013
			0.8	566	0.8988	0.9002	–0.0014
		0.10	0.6	100	0.9039	0.9029	0.0010
			0.7	256	0.9033	0.9012	0.0021
			0.8	1170	0.8999	0.9000	–0.0001

Furthermore, under the prescribed model configurations, simulation study was conducted to justify the accuracy of the proposed power and sample size procedures. Specifically, the simulated power of the proposed test procedure was computed via Monte Carlo simulation of 10,000 independent data sets. The simulated power and the difference between the simulated power and estimated power are also presented in Table 6. For each of the 18 scenarios, the small difference reveals that the simulated power is nearly identical to the estimated power. The accuracy of the described power and sample size procedures is fairly consistent under various sample size and parameter configurations. Consequently, these findings suggest that the developed power and sample size algorithms are reliable for practical applications.

### An application

A bioequivalence study was presented in Liu and Chow [14] to demonstrate the assessment of individual equivalence between two drug formulations. Under the standard setting of two-sequence two-period cross over design, the responses are the area under the plasma concentration-time curve (AUC). The sample sizes, sample mean difference, and residual error variance of the logarithmic transformation of AUC are *N*_1_ = *N*_2_ = 10, ${\bar{C}}_{1}$ – ${\bar{C}}_{2}$ = 0.05331, and *S*^2^ = 0.0378, respectively. To declare individual equivalence between the test and reference formulations, it is assumed that at least p* = 0.75 of the difference between two individual formulation measurements are within the bounds Δ_*L*_ = *ln*(0.80) = –0.2231 and Δ_*U*_ = *ln*(1.25) = 0.2231. Accordingly, the test statistics in Eq 21 can be computed as *T*_*CL*_ = 3.1801 and *T*_*CU*_ = –1.9537. With α = 0.05, the critical values of the TOST and proposed procedures are τ_*CT*_ = 6.0173 and τ_*CE*_ = 4.3436, respectively. Also, the two associated critical regions are (${\hat{\theta }}_{CTL}$, ${\hat{\theta }}_{CTU}$) = (–0.4698, 0.5764) and (${\hat{\theta }}_{CEL}$, ${\hat{\theta }}_{CEU}$) = (–0.3243, 0.4309). Thus, the two test procedures conclude that the null hypothesis of no individual equivalence cannot be rejected at the significance level 0.05.

Under the normal assumptions, the difference between two individual formulation measurements has the distribution (*C*_1*k*_–*C*_2*k′*_) ~ *N*(μ_*C*_, $\sigma_{C}^{2}$). Using the summary statistics as exemplifying parameter values (μ_*C*_, $\sigma_{C}^{2}$) = (0.05331, 0.0756), the proportion between the two bounds (Δ_*L*_, Δ_*U*_) = (–0.2231, 0.2231) for the normal distribution *N*(μ_*C*_, $\sigma_{C}^{2}$) is the probability *P*(Δ_*L*_ < *C*_1*k*_–*C*_2*k′*_ < Δ_*U*_) = 0.5744. Note that the coverage probability is substantially less than the nominal value 0.75 for declaring individual equivalence. For illustration, the working parameters are chosen as μ_*C*_ = 0.02, 0.03, 0.04, and 0.05 and $\sigma_{C}^{2}$ = 0.0756/4. To meet the nominal power 0.80, the estimated sample sizes are (*N*_1_, *N*_2_) = (25, 25), (37, 37), (69, 69), and (183, 183) with the achieved power levels 0.8017, 0.8035, 0.8024, and 0.8002, respectively. Evidently, the magnitudes are larger than the sample sizes (*N*_1_, *N*_2_) = (10, 10) of the previous analysis. This indicates the importance and accuracy of power and sample size procedures for efficient computations in individual equivalence study. The accompanying computer algorithms are also presented for conducting the suggested power and sample size calculations.

## Conclusions

The conventional TOST of mean focuses only on the equivalence of population means between the test and reference formulations. Therefore, the TOST of mean equivalence or average equivalence does not take into account the variability of formulation difference in bioavailability across subjects. In view of the limitation of average equivalence, Chen [24] identified several desirable features of bioequivalence criteria. The criteria include the assurance of switchability between formulations, the control of Type I error rate at 5%, determination of appropriate sample size, and user-friendly software application for the statistical method. Related considerations of individual equivalence can be found in the additional discussion in Chen et al. [25] and Chen and Lesko [26]. To address these issues, this article presents exact tests for assessing individual equivalence under parallel group and crossover designs. The numerical results showed that the TOST procedures based on tolerance intervals are overly conservative. More importantly, the exact approach has excellent Type I error control and can be recommended for routine use. Computer programs are also developed to implement the proposed equivalence test, power calculation, and sample size determination. The research designs and test procedures considered here are valid only if the homogeneous variance assumption is satisfied. The degree of robustness presumably depends on the extent of how badly the homogeneity of variance assumption is violated. Future research can explore possible extensions to accommodate heterogeneity of variance settings.

## Supporting information

S1 File SAS/IML programs for performing the suggested procedures. (PDF)

S2 File R programs for performing the suggested procedures. (PDF)

## References

[1] Schuirmann D. L. (1981). On hypothesis testing to determine if the mean of a normal distribution is contained in a known interval. Biometrics, 37, 617.

[2] Westlake W. J. (1981). Response to T.B.L. Kirkwood: Bioequivalence testing |a need to rethink. Biometrics, 3, 589–594.

[3] Meyners M. (2012). Equivalence tests-A review. Food Quality and Preference, 26, 231–245.

[4] Hauschke D., Steinijans V., & Pigeot I. (2007). Bioequivalence studies in drug development: Methods and applications. Chichester: John Wiley & Sons.

[5] Chow S. C., & Liu J. P. (2008). Design and analysis of bioavailability and bioequivalence studies (3rd ed.). New York, NY: Chapman & Hall/CRC.

[6] Wellek S. (2010). Testing statistical hypotheses of equivalence and noninferiority (2nd ed.). New York, NY: CRC Press.

[7] Choudhary P. K., & Nagaraja H. N. (2017). Measuring agreement: Models, methods, and applications. Hoboken, NJ: John Wiley & Sons.

[8] Anderson S., & Hauck W. W. (1990). Consideration of individual bioequivalence. Journal of Pharmacokinetics and Biopharmaceutics, 18, 259–273. doi: 10.1007/BF01062202 2380920

[9] Hauck W. W., & Anderson S. (1992). Types of bioequivalence and related statistical considerations. International Journal of Clinical Pharmacology, Therapy and Toxicology, 30, 181–187. 1592546

[10] Sheiner L. B. (1992). Bioequivalence revisited. Statistics in Medicine, 11, 1777–1788. doi: 10.1002/sim.4780111311 1485060

[11] Schall R., & Luus G. H. (1993). On population and individual bioequivalence. Statistics in Medicine, 12, 1109–1124. doi: 10.1002/sim.4780121202 8210816

[12] Anderson S. (1993). Individual bioequivalence: A problem of switchability (with discussion). Biopharmaceutical Report, 2, 1–11.

[13] Esinhart J. D., & Chinchilli V. M. (1994). Extension to the use of tolerance intervals for the assessment of individual bioequivalence. Journal of Biopharmaceutical Statistics, 4, 39–52. doi: 10.1080/10543409408835071 8019583

[14] Liu J. P., & Chow S. C. (1997). A two one-sided tests procedure for assessment of individual bioequivalence. Journal of Biopharmaceutical Statistics, 7, 49–61. doi: 10.1080/10543409708835169 9056588

[15] Tsong Y., & Shen M. (2007). An alternative approach to assess exchangeability of a test treatment and the standard treatment with normally distributed response. Journal of Biopharmaceutical Statistics, 17, 329–338. doi: 10.1080/10543400601177301 17365227

[16] Krishnamoorthy K., & Mathew T. (2009). Statistical tolerance regions: Theory, applications, and computation (Vol. 744). New York, NY: Wiley.

[17] Meeker W. Q., Hahn G. J., & Escobar L. A. (2017). Statistical intervals: A guide for practitioners and researchers. Hoboken, NJ: Wiley.

[18] Berger R. L., & Hsu J. C. (1996). Bioequivalence trials, intersection-union tests and equivalence confidence sets (with discussion). Statistical Science, 11, 283–319.

[19] Owen D. B. (1965). A special case of a bivariate non-central t-distribution. Biometrika, 52, 437–446.

[20] Shieh G. (2020). Comparison of alternative approaches for difference, noninferiority, and equivalence testing of normal percentiles. BMC Medical Research Methodology, 20, 59. doi: 10.1186/s12874-020-00933-z 32169043 PMC7071592

[21] Johnson N. L., Kotz S., & Balakrishnan N. (1995). Continuous univariate distributions (2nd ed., Vol. 2). New York, NY: Wiley.

[22] Hahn G. J. (1970). Statistical intervals for a normal population, Part I. Tables, examples and applications. Journal of Quality Technology, 2, 115–125.

[23] Hahn G. J. (1970). Statistical intervals for a normal population, Part II. Formulas, assumptions, some derivations. Journal of Quality Technology, 2, 195–206.

[24] Chen M. L. (1997). Individual bioequivalence-A regulatory update. Journal of Biopharmaceutical Statistics, 7, 5–11. doi: 10.1080/10543409708835162 9056581

[25] Chen M. L., Patnaik R., Hauck W. W., Schuirmann D. J., Hyslop T., Williams R. (2000). An individual bioequivalence criterion: Regulatory considerations. Statistics in Medicine, 19, 2821–2842. doi: 10.1002/1097-0258(20001030)19:20<2821::aid-sim548>3.0.co;2-l 11033578

[26] Chen M. L., & Lesko L. J. (2001). Individual bioequivalence revisited. Clinical pharmacokinetics, 40, 701–706. doi: 10.2165/00003088-200140100-00001 11707058

