# Supplementary material for: Assessing individual equivalence in parallel group and crossover designs: Exact test and sample size procedures
Source: PLoS One. 2022 May 27;17(5):e0269128. doi: 10.1371/journal.pone.0269128 (PMC9140302; doi:10.1371/journal.pone.0269128)
Supplement: S1 File — (PDF) [file pone.0269128.s001.pdf]

Program S1

SAS/IML program for conducting individual equivalence test

```
PROC IML;
*USER SPECIFICATION PORTION;
*DESIGNATED ALPHA;ALPHA=0.05;
*NULL CENTRAL PROPORTION;PROP=0.75;
*EQUIVALENCE BOUNDS;DELTAL=LOG(0.8);DELTAU=LOG(1.25);
*SAMPLE SIZES;N1=10;N2=10;
*SAMPLE MEAN DIFFERENCE;MED=0.05331;
*SAMPLE VARIANCE;S2=0.0378;
*END OF USER SPECIFICATION PORTION;

PCT=(1+PROP)/2;ZP=QUANTILE('NORMAL',PCT);
DF=N1+N2-2;M=1/(1/N1+1/N2);S=SQRT(S2);
STDH=S/SQRT(M);
PRINT ALPHA PROP PCT ZP[FORMAT=8.4] DELTAL[FORMAT=8.4]
DELTAU[FORMAT=8.4];
PRINT N1 N2 MED S2[FORMAT=10.6] S[FORMAT=8.4];

NUMINT=1000;
COEVECC=({1}||REPEAT({4 2},1,NUMINT/2-1)||{4 1})`;
CL=10E-8;CU=QUANTILE('CHISQ',1-CL,DF);
INT=CU-CL;INTL=INT/NUMINT;
CVEC=CL+(INTL#(0:NUMINT))`;
WCPDF=(INTL/3)#COEVECC#PDF('CHISQ',CVEC,DF);
START FUNTAUE;
TAUEL=0;TAUEU=1000;
DO UNTIL(ABS(DALPHA)<10E-9 & DALPHA>0);
TAUET=(TAUEL+TAUEU)/2;
G=ZP#SQRT(2#M)-TAUET#SQRT(CVEC/DF);
K0=2#DF#M#(ZP/TAUET)##2;
G0=G#(CVEC<K0);
ALPHAT=WCPDF`*(2#CDF('NORMAL',G0)-1);
IF ALPHAT<ALPHA THEN TAUEU=TAUET;ELSE TAUEL=TAUET;
DALPHA=ALPHAT-ALPHA;END;
TAUE=TAUET;
FINISH;
```

```
RUN FUNTAUE;
TL=(MED-DELTAL)/STDH;TU=(MED-DELTAU)/STDH;
TESTE=(TAUE<TL)#(TU<-TAUE);
THETAEL=MED-TAUE#STDH;THETAEU=MED+TAUE#STDH;
PRINT "EXACT TEST:" TL[FORMAT=8.4] TU[FORMAT=8.4]
TAUE[FORMAT=8.4];
PRINT THETAEL[FORMAT=8.4] THETAEU[FORMAT=8.4];
IF TESTE=1 THEN PRINT "REJECT H0";ELSE PRINT "DON'T REJECT H0";
QUIT;
```

Program S2

SAS/IML program for computing attained power for individual equivalence test

```
PROC IML;
*USER SPECIFICATION PORTION;
*DESIGNATED ALPHA;ALPHA=0.05;
*NULL CENTRAL PROPORTION;PROP=0.75;
*EQUIVALENCE BOUNDS;DELTAL=LOG(0.8);DELTAU=LOG(1.25);
*SAMPLE SIZES;N1=25;N2=25;
*MEAN DIFFERENCE;MUD=0.02;
*ERROR VARIANCE;SIGSQ=0.0756/8;
*END OF USER SPECIFICATION PORTION;

DF=N1+N2-2;M=1/(1/N1+1/N2);NT=N1+N2;
PCT=(1+PROP)/2;ZP=QUANTILE('NORMAL',PCT);
MUD0=(DELTAL+DELTAU)/2;SIGMAD0=(DELTAU-DELTAL)/(2#ZP);
SIGSQD0=SIGMAD0##2;SIGSQ0=SIGSQD0/2;SIGMA0=SQRT(SIGSQ0);
PRINT ALPHA MUD0 PROP PCT ZP[FORMAT=8.4] DELTAL[FORMAT=8.4]
DELTAU[FORMAT=8.4];
PRINT SIGMA0[FORMAT=8.4] SIGSQ0[FORMAT=8.4] SIGMAD0[FORMAT=8.4]
SIGSQD0[FORMAT=8.4];

SIGSQD=2#SIGSQ;SIGMAD=SQRT(SIGSQD);SIGMA=SQRT(SIGSQ);
PROP1=CDF('NORMAL',(DELTAU-MUD)/SIGMAD)-CDF('NORMAL',(DELTAL-M
UD)/SIGMAD);
THETAL=MUD-ZP#SIGMAD;THETAU=MUD+ZP#SIGMAD;
PRINT MUD SIGSQ[FORMAT=8.4] SIGSQD[FORMAT=8.4] PROP1[FORMAT=8.4]
THETAL[FORMAT=8.4] THETAU[FORMAT=8.4];

NUMINT=1000;
COEVECC=({1}||REPEAT({4 2},1,NUMINT/2-1)||{4 1})`;
CL=10E-8;CU=QUANTILE('CHISQ',1-CL,DF);
INT=CU-CL;INTL=INT/NUMINT;
CVEC=CL+(INTL#(0:NUMINT))`;
WCPDF=(INTL/3)#COEVECC#PDF('CHISQ',CVEC,DF);
START FUNTAUE;
TAUEL=0;TAUEU=1000;
DO UNTIL(ABS(DALPHA)<10E-9 & DALPHA>0);
TAUET=(TAUEL+TAUEU)/2;
```

```
G=ZP#SQRT(2#M)-TAUET#SQRT(CVEC/DF);
K0=2#DF#M#(ZP/TAUET)##2;
G0=G#(CVEC<K0);
ALPHAT=WCPDF`*(2#CDF('NORMAL',G0)-1);
IF ALPHAT<ALPHA THEN TAUEU=TAUET;ELSE TAUEL=TAUET;
DALPHA=ALPHAT-ALPHA;END;
TAUE=TAUET;
FINISH;

STD=SQRT(SIGSQ/M);RUN FUNTAUE;
GL=(DELTAL-MUD)/STD+TAUE#SQRT(CVEC/DF);
GU=(DELTAU-MUD)/STD-TAUE#SQRT(CVEC/DF);
K1=(DF#M#(DELTAU-DELTAL)##2)/(4#SIGSQ#TAUE##2);
GL1=GL#(CVEC<K1);GU1=GU#(CVEC<K1);
EPOWER=WCPDF`*(CDF('NORMAL',GU1)-CDF('NORMAL',GL1));
PRINT TAUE[FORMAT=8.4] EPOWER[FORMAT=8.4] N1 N2 NT;
QUIT;
```

Program S3

SAS/IML program for computing required sample size for individual equivalence test

```
PROC IML;
*USER SPECIFICATION PORTION;
*DESIGNATED ALPHA;ALPHA=0.05;
*NOMINAL POWER;POWER=0.80;
*NULL CENTRAL PROPORTION;PROP=0.75;
*EQUIVALENCE BOUNDS;DELTAL=LOG(0.8);DELTAU=LOG(1.25);
*MEAN DIFFERENCE;MUD=0.02;
*ERROR VARIANCE;SIGSQ=0.0756/8;
*SAMPLE SIZE RATIO;R21=1;
*END OF USER SPECIFICATION PORTION;

PCT=(1+PROP)/2;ZP=QUANTILE('NORMAL',PCT);
MUD0=(DELTAL+DELTAU)/2;SIGMAD0=(DELTAU-DELTAL)/(2#ZP);
SIGSQD0=SIGMAD0##2;SIGSQ0=SIGSQD0/2;SIGMA0=SQRT(SIGSQ0);
PRINT ALPHA MUD0 PROP PCT ZP[FORMAT=8.4] DELTAL[FORMAT=8.4]
DELTAU[FORMAT=8.4] POWER;
PRINT SIGMA0[FORMAT=8.4] SIGSQ0[FORMAT=8.4] SIGMAD0[FORMAT=8.4]
SIGSQD0[FORMAT=8.4];

SIGSQD=2#SIGSQ;SIGMAD=SQRT(SIGSQD);SIGMA=SQRT(SIGSQ);
PROP1=CDF('NORMAL',(DELTAU-MUD)/SIGMAD)-CDF('NORMAL',(DELTAL-M
UD)/SIGMAD);
THETAL=MUD-ZP#SIGMAD;THETAU=MUD+ZP#SIGMAD;
PRINT MUD SIGSQ[FORMAT=8.4] SIGSQD[FORMAT=8.4] PROP1[FORMAT=8.4]
THETAL[FORMAT=8.4] THETAU[FORMAT=8.4];

NUMINT=1000;
COEVECC=({1}||REPEAT({4 2},1,NUMINT/2-1)||{4 1})`;CL=10E-8;
START FUNTAUE;
TAUEL=0;TAUEU=1000;
DO UNTIL(ABS(DALPHA)<10E-9 & DALPHA>0);
TAUET=(TAUEL+TAUEU)/2;
G=ZP#SQRT(2#M)-TAUET#SQRT(CVEC/DF);
K0=2#DF#M#(ZP/TAUET)##2;
G0=G#(CVEC<K0);
ALPHAT=WCPDF`*(2#CDF('NORMAL',G0)-1);
```

```
IF ALPHAT<ALPHA THEN TAUEU=TAUET;ELSE TAUEL=TAUET;
DALPHA=ALPHAT-ALPHA;END;
TAUE=TAUET;
FINISH;

N1=5;
DO UNTIL(EPOWER>POWER | N1>1000);
N1=N1+1;N2=R21#N1;DF=N1+N2-2;M=1/(1/N1+1/N2);
CU=QUANTILE('CHISQ',1-CL,DF);
INT=CU-CL;INTL=INT/NUMINT;
CVEC=CL+(INTL#(0:NUMINT))`;
WCPDF=(INTL/3)#COEVECC#PDF('CHISQ',CVEC,DF);
STD=SQRT(SIGSQ/M);RUN FUNTAUE;
GL=(DELTAL-MUD)/STD+TAUE#SQRT(CVEC/DF);
GU=(DELTAU-MUD)/STD-TAUE#SQRT(CVEC/DF);
K1=(DF#M#(DELTAU-DELTAL)##2)/(4#SIGSQ#TAUE##2);
GL1=GL#(CVEC<K1);GU1=GU#(CVEC<K1);
EPOWER=WCPDF`*(CDF('NORMAL',GU1)-CDF('NORMAL',GL1));
END;NT=N1+N2;
PRINT TAUE[FORMAT=8.4] EPOWER[FORMAT=8.4] N1 N2 NT;
QUIT;
```
