# Supplementary material for: Assessing individual equivalence in parallel group and crossover designs: Exact test and sample size procedures
Source: PLoS One. 2022 May 27;17(5):e0269128. doi: 10.1371/journal.pone.0269128 (PMC9140302; doi:10.1371/journal.pone.0269128)
Supplement: S2 File — (PDF) [file pone.0269128.s002.pdf]

Program R1
R program for conducting individual equivalence test

```
function () {#prl.homt.roaht.apx1
#USER SPECIFICATIONS PORTION
alpha=0.05 #DESIGNATED ALPHA
prop=0.75 #NULL CENTRAL PROPORTION
deltal=log(0.8)
deltau=log(1.25) #EQUIVALENCE BOUNDS
n1=10
n2=10 #SAMPLE SIZES
med<-0.05331 #SAMPLE MEAN DIFFERENCE
s2<-0.0378 #SAMPLE VARIANCE
#END OF SPECIFICATION

pct=(1+prop)/2
zp=qnorm(pct)
df=n1+n1-2
m=1/(1/n1+1/n2)
s=sqrt(s2)
stdh=s/sqrt(m)
print(c("alpha,prop,pct,zp,deltal,deltau"))
print(c(alpha,prop,pct,zp,deltal,deltau))
print(c("n1,n2,med,s2,s"))
print(c(n1,n2,med,s2,s))

numint=1000
coevec=c(1,rep(c(4,2),numint/2-1),4,1)
cl=1e-6
funtaue=function () {
tauel=0
taueu=1000
dalpha=1
while(abs(dalpha)>1e-9 | dalpha>0){
tauet=(tauel+taueu)/2
g=zp*sqrt(2*m)-tauet*sqrt(cvec/df)
k0=2*df*m*(zp/tauet)^2
g0=g*(cvec<k0)
alphat=sum(wcpdf*(2*pnorm(g0)-1))
```

```
if (alphat<alpha) taueu=tauet else tauel=tauet
dalpha=alphat-alpha}
taue=tauet
return(taue)}

cu=qchisq(1-cl,df)
int=cu-cl
intl=int/numint
cvec=cl+intl*(0:numint)
wcpdf=(intl/3)*coevec*dchisq(cvec,df)
taue=funtaue()
tl=(med-deltal)/stdh
tu=(med-deltau)/stdh
teste=(taue < tl)*(tu < -taue)
thetal=med-taue*stdh
thetau=med+taue*stdh
print(c("tl,tu,taue"))
print(c(tl,tu,taue))
print(c("thetal,thetau"))
print(c(thetal,thetau))
if (teste==1) test=c("reject ho") else test=("don't reject H0")
print(c("test:",test))}
```

Program R2

R program for computing attained power for individual equivalence test

```
function () {#prl.homt.roaht.apx2
#USER SPECIFICATIONS PORTION
alpha=0.05 #DESIGNATED ALPHA
prop=0.75 #NULL CENTRAL PROPORTION
deltal=log(0.8)
deltau=log(1.25) #EQUIVALENCE BOUNDS
n1=25
n2=25 #SAMPLE SIZES
mud<-0.02 #MEAN DIFFERENCE
sigsq<-0.0756/8 #ERROR VARIANCE
#END OF SPECIFICATION

pct=(1+prop)/2
zp=qnorm(pct)
df=n1+n1-2
m=1/(1/n1+1/n2)
nt=n1+n2
mud0=(deltal+deltau)/2
sigmad0=(deltau-deltal)/(2*zp)
sigsqd0=sigmad0^2
sigsq0=sigsqd0/2
sigma0=sqrt(sigsq0)
print(c("alpha,mud0,prop,pct,zp,deltal,deltau"))
print(c(alpha,mud0,prop,pct,zp,deltal,deltau))
print(c("sigma0,sigsq0,sigmad0,sigsqd0"))
print(c(sigma0,sigsq0,sigmad0,sigsqd0))

sigsqd=2*sigsq
sigmad=sqrt(sigsqd)
sigma=sqrt(sigsq)
prop1=pnorm((deltau-mud)/sigmad)-pnorm((deltal-mud)/sigmad)
thetal=mud-zp*sigmad
thetau=mud+zp*sigmad
print(c("mud,sigsq,sigsqd,prop1,thetal,thetau"))
print(c(mud,sigsq,sigsqd,prop1,thetal,thetau))
```

```
numint=1000
coevec=c(1,rep(c(4,2),numint/2-1),4,1)
cl=1e-6
funtaue=function () {
tauel=0
taueu=1000
dalpha=1
while(abs(dalpha)>1e-9 | dalpha>0){
tauet=(tauel+taueu)/2
g=zp*sqrt(2*m)-tauet*sqrt(cvec/df)
k0=2*df*m*(zp/tauet)^2
g0=g*(cvec<k0)
alphat=sum(wcpdf*(2*pnorm(g0)-1))
if (alphat<alpha) taueu=tauet else tauel=tauet
dalpha=alphat-alpha}
taue=tauet
return(taue)}

cu=qchisq(1-cl,df)
int=cu-cl
intl=int/numint
cvec=cl+intl*(0:numint)
wcpdf=(intl/3)*coevec*dchisq(cvec,df)
taue=funtaue()
std=sqrt(sigsq/m)
gl=(deltal-mud)/std+taue*sqrt(cvec/df)
gu=(deltau-mud)/std-taue*sqrt(cvec/df)
k1=(df*m*(deltau-deltal)^2)/(4*sigsq*taue^2)
gl1=gl*(cvec<k1)
gu1=gu*(cvec<k1)
epower=sum(wcpdf*(pnorm(gu1)-pnorm(gl1)))
print(c("taue,epower,n1,n2,nt"))
print(c(taue,epower,n1,n2,nt))}
```

Program R3

R program for computing required sample size for individual equivalence test

```
function () {#prl.homt.roaht.apx3
#USER SPECIFICATIONS PORTION
alpha=0.05 #DESIGNATED ALPHA
power=0.8 #NOMINAL POWER
prop=0.75 #NULL CENTRAL PROPORTION
deltal=log(0.8)
deltau=log(1.25) #EQUIVALENCE BOUNDS
mud<-0.02 #MEAN DIFFERENCE
sigsq<-0.0756/8 #ERROR VARIANCE
r21=1 #SAMPLE SIZE RATIO
#END OF SPECIFICATION

pct=(1+prop)/2
zp=qnorm(pct)
mud0=(deltal+deltau)/2
sigmad0=(deltau-deltal)/(2*zp)
sigsqd0=sigmad0^2
sigsq0=sigsqd0/2
sigma0=sqrt(sigsq0)
print(c("alpha,mud0,prop,pct,zp,deltal,deltau,power"))
print(c(alpha,mud0,prop,pct,zp,deltal,deltau,power))
print(c("sigma0,sigsq0,sigmad0,sigsqd0"))
print(c(sigma0,sigsq0,sigmad0,sigsqd0))

sigsqd=2*sigsq
sigmad=sqrt(sigsqd)
sigma=sqrt(sigsq)
prop1=pnorm((deltau-mud)/sigmad)-pnorm((deltal-mud)/sigmad)
thetal=mud-zp*sigmad
thetau=mud+zp*sigmad
print(c("mud,sigsq,sigsqd,prop1,thetal,thetau"))
print(c(mud,sigsq,sigsqd,prop1,thetal,thetau))

numint=1000
coevec=c(1,rep(c(4,2),numint/2-1),4,1)
cl=1e-6
```

```
funtaue=function () {
tauel=0
taueu=1000
dalpha=1
while(abs(dalpha)>1e-9 | dalpha>0){
tauet=(tauel+taueu)/2
g=zp*sqrt(2*m)-tauet*sqrt(cvec/df)
k0=2*df*m*(zp/tauet)^2
g0=g*(cvec<k0)
alphat=sum(wcpdf*(2*pnorm(g0)-1))
if (alphat<alpha) taueu=tauet else tauel=tauet
dalpha=alphat-alpha}
taue=tauet
return(taue)}

n1=5
epower=0
while(epower<power & n1<1000){
n1=n1+1
n2=n1*r21
df=n1+n2-2
m=1/(1/n1+1/n2)
cu=qchisq(1-cl,df)
int=cu-cl
intl=int/numint
cvec=cl+intl*(0:numint)
wcpdf=(intl/3)*coevec*dchisq(cvec,df)
taue=funtaue()
std=sqrt(sigsq/m)
gl=(deltal-mud)/std+taue*sqrt(cvec/df)
gu=(deltau-mud)/std-taue*sqrt(cvec/df)
k1=(df*m*(deltau-deltal)^2)/(4*sigsq*taue^2)
gl1=gl*(cvec<k1)
gu1=gu*(cvec<k1)
epower=sum(wcpdf*(pnorm(gu1)-pnorm(gl1)))}
nt=n1+n2
print(c("taue,epower,n1,n2,nt"))
print(c(taue,epower,n1,n2,nt))}
```
